# Trajectories of Personal Well-Being Attributes Among High School Students in Hong Kong

**DOI:** 10.1007/s11482-016-9492-5

**Published:** 2016-10-27

**Authors:** Daniel T. L. Shek, Li Lin

**Affiliations:** Department of Applied Social Sciences, The Hong Kong Polytechnic University, Hong Kong, Hong Kong

**Keywords:** Personal well-being, Positive youth development, Developmental trajectories, Hong Kong adolescents

## Abstract

How personal well-being of adolescents changes over the high school period is not entirely clear in the scientific literature. Using 15 positive youth development (PYD) attributes as indicators of well-being, the current study investigated the related developmental trajectories among a large sample of Hong Kong adolescents from Grade 7 to Grade 12. Individual growth curve modeling revealed that while variation existed across different PYD attributes, nine out of 15 positive youth development attributes declined in the early adolescence but showed a rebound or a slower rate of decline in the late adolescence. The current study serves as a pioneering attempt to chart the normative profiles of Chinese adolescents’ personal well-being over time. The theoretical and practical implications of the findings are discussed.

## Introduction

Adolescence was traditionally portrayed as a period of “storm and stress” (Hall 1904). It is believed that young people typically undergo considerable changes in family, school, peer, and individual domains during adolescence, which put them at heightened risk of increased mood disruptions, problem behavior and conflict with parents (Arnett 1999). The general hypothesis derived from this view is that personal well-being of adolescents would deteriorate during adolescent years. In contrast to the “storm and stress” notion, there are views suggesting that adolescents are able to develop their strengths, maintain their personal well-being, and finally navigate through the adolescent period successfully (Lerner et al. 2013). The proposal that youths thrive in adolescent years can be seen in the positive youth development approach (PYD approach), an alternative approach that regards adolescents as “resource to be developed” and believes in their relative plasticity (Lerner 2004; Roth and Brooks-Gunn 2003).

Empirically, there are research findings showing the decline in the positive dispositions or behavior when youths transit into adolescence, such as dampened self-concept (e.g., Wigfield et al. 1991) and declined academic engagement (Wang and Eccles 2012). Nevertheless, mounting evidence has also suggested that the “storm and stress” notion does not apply to all the adolescents (Arnett 1999; Hollenstein and Lougheed, 2013). In fact, most of the adolescents did not experience dramatic decline in adaptive functioning and personal well-being during adolescence (e.g., Birkeland et al. 2012; Warren et al. 2016). Studies underlying the PYD approach have revealed a more “optimistic” developmental trajectory of adaptive functioning and personal well-being over adolescent years where after the initial decline there is a rebound of some positive functioning such as positive identity (Shek and Ma 2012) or where there is constant growth in some positive functioning, such as persistence, self-discipline, and regulation (Hilliard et al. 2014). In the area of adolescent risk behavior, there are also studies showing that while adolescent risk behavior begins to grow in the early adolescence, it slows down as time goes by (e.g., Farrell et al. 2005; Shek and Yu 2012).

However, studies examining the developmental trajectories of PYD attributes are far from adequate. The majority of the studies (e.g., Geldhof et al. 2014; Hilliard et al. 2014) were based on the US samples derived from the 4-H project (Bowers et al. 2015). To better understand how adolescents develop their PYD attributes, it is imperative to replicate and extend the previous findings with other samples and with more PYD attributes (e.g., Warren et al. 2016). As such, in this study based on the framework of Catalano et al. (2004), we investigated the developmental trajectories of PYD attributes among a group of Hong Kong adolescents from Grade 7 to Grade 12.

### The Positive Youth Development (PYD) Approach

The positive youth development approach is derived from developmental systems theory, which emphasizes the developmental regulation between individual and context (Lerner 2004). The PYD approach upholds a belief that every adolescent has the potential to thrive. When the interaction between individuals and contexts is mutually beneficial, positive youth development is achieved, which is reflected by higher levels of competence (i.e., positive view of one’s ability), confidence (a sense of overall positive self-regard), connection (i.e., positive bonds with people and institutions), character (i.e., respect for social rules; a sense of right and wrong), and caring (i.e., sympathy and empathy for others). These indicators of PYD were termed as “Five 5Cs” according to Lerner et al. (2013).

Theoretically, PYD is an indicator of personal well-being (Shek and Lin 2014). The traditional conception of psychological well-being primarily concerns hedonic well-being, including experience of happiness and absence of pain (Kubovy et al. 1999). However, the eudaimonic view considers psychological well-being more than that. It incorporates positive human functioning and psychological growth. For example, Ryff and Keyes (1995) proposed that psychological well-being consists of six distinct aspects of human actualization, including autonomy, self-acceptance, personal growth, mastery, life purpose, and positive relatedness. Ryan and Deci (2001) interpreted psychological well-being as self-actualization and vitality. According to these views, PYD entails positive youth functioning which reflects adolescents’ personal well-being.

Through decades of research, advocates of the PYD approach have consensus on several issues regarding youth development (e.g., Lerner 2004; Phelps et al. 2007). First, different from the “storm and stress” hypothesis, most adolescents maintain relatively high levels of adaptive functioning notwithstanding fluctuation over time, and some adolescents in fact do not experience decline in general PYD over the high school years (e.g., Lewin-Bizan et al. 2010).

Second, the degree of positive development depends on the regulation within the interaction between the individual and developmental contexts. In the transition from primary school to high school, adolescents might experience a mismatch between their developmental needs (e.g., increasing need for autonomy) and the environment (e.g., controlling context in school or family; Eccles et al. 1993). This poses challenges to their positive functioning, and thus negative changes of PYD attributes occur in early high school years (Geldhof et al. 2014). However, adolescents have relative plasticity, which implies that adolescents’ positive functioning could be dampened if the contexts fail to match and support their developmental needs, while it can also be enhanced if individuals are able to fit in the contexts. The idea of the relative plasticity of human development denotes an optimistic message that every youth has the potential to thrive. This view is in line with Erikson (1968)’s theory of psychosocial development. Even though life crisis such as identity crisis exists in adolescence, the turmoil is not a “must” across all individuals and over the entire adolescent period. There are four forms of adolescent identity: identity diffusion, foreclosure, moratorium and identity achievement (Marcia 1980). While turmoil exists in adolescents with “identity diffusion” or “identity moratorium,” adolescents with “identity achievement” status usually have a good well-being after struggling through the moratorium stage (i.e., a U shape in positive development).

Finally, diversity exists in the developmental course. Diversity refers to changes in the course of intrapersonal development, as well as the individual differences in the developmental trajectories. The PYD approach regards diversity as “a strength of individuals and an asset for planning and promoting means to improve the human condition” (Lerner 2004, p.336). It implies the possibility to modify one’s developmental pattern within his/her life-span.

### Empirical Studies on Change in PYD during Adolescence

There are only limited empirical research findings in this area. The majority of the findings have been generated from the 4-H study, which was launched in 2002 with the support of the National 4-H Council in the US (Bowers et al. 2015). The 4-H study included eight waves of data collection among adolescents and parents, and repeatedly assessed the Five Cs of PYD (i.e., competence, confidence, connection, character, and caring; Bowers et al. 2010) as well as their contribution (the sixth C as an outcome of PYD; Hershberg et al. 2015). The developmental trajectories of these variables have been investigated using either variable-centered or person-centered approach (see Tables 1 and 2 for a summary). Using the sample tracked from Grade 5 to Grade 7, Phelps et al. (2007) found that the overall PYD (the composite score of Five Cs) decreased, while the decrease from Grade 5 to Grade 6 was greater than that from Grade 6 to Grade 7. The researchers also identified five kinds of trajectories of overall PYD among the participants. While 14 % of the adolescents experienced a decline in PYD, 17.4 % experienced an increase. The rest remained stable at high (28.79 %), medium (35.35 %) or low levels (4.45 %). When the assessment period was extended from Grade 5 to Grade 10, Lewin-Bizan et al. (2010) found four kinds of trajectories, with more than half of the adolescents experiencing an increase to a stable-moderate level (39 %) or a stable-high level (28.3 %). Although some of them (26.5 %) showed an increase followed by a decrease in PYD, only a small proportion (6.2 %) showed a continuous decrease in PYD. By looking into the specific constructs, Geldhof et al. (2014) found that from Grade 5 to Grade 12, competence, confidence, and connection on average declined during the early high school years while character and caring remained stable. Additionally, Hilliard et al. (2014)’s study revealed that youth contribution (i.e., leadership, service, helping behavior, and ideology about the importance of contribution) was on the rise with the increase rate becoming faster from Grade 7 to Grade 12.

**Table 1:** Summary of studies on developmental trajectories of PYD attributes using variable-centered approach

Author (publication Year)	Age	Findings
1. Phelps et al. (2007)	Grade 5 to Grade 7	PYD: decrease, decrease slower
2. Geldhof et al. (2014)	Grade 5 to Grade 12	PYD: constant Character/Caring: constant Competence/Confidence/Connection: decline in early high school years
3. Hilliard et al. (2014)	Grade 7 to Grade 10	Civic Character (contribution): increase, increase faster Performance character: increase, increase faster Moral character (behavioral conduct): no change

**Table 2:** Summary of studies on developmental trajectories of PYD attributes using person-centered approach

Author (publication Year)	Age	Findings
1. Phelps et al. (2007)	Grade 5 to Grade 7	PYD: five trajectories were identified a. Low-increase (17.4 %) b. medium-decrease (14.0 %) c. high-constant (28.79 %) d. medium-constant (35.35 %) e. low-constant (4.45 %)
2. Zimmerman et al. (2008)	Grade 5 to Grade 8	PYD: five trajectories were identified a. low-increase (21.3 %) b. low-decrease (3.7 %) c. high-constant (27.6 %) d. medium-constant (39.6 %) e. low-constant (7.8 %) Contribution: three trajectories were identified a. high-constant (27.4 %) b. low-increase (59.2 %) c. low-constant (13.3 %)
3. Lewin-Bizan et al. (2010)	Grade 5 to Grade 10	PYD: four trajectories were identified a. increase to stable high (28.3 %) b. increase to stable moderate (39.0 %) c. increase, decrease (26.5 %) d. decrease (6.2 %) Contribution: four trajectories were identified a. increase to stable high (26.9 %) b. increase to stable moderate (52.6 %) c. high, increase (7.0 %) d. low, constant (13.5 %)
4. Schmid et al. (2011)	Grade 7 to Grade 9	PYD: four trajectories were identified a. very high-constant (24 %) b. high-constant (42 %) c. medium-constant (27 %) d. low-decrease (7 %)

Research on PYD trajectory outside the US is rare. However, Shek and his colleagues’ work on Chinese adolescents in Hong Kong is an outstanding exception. In 2005, Shek and his colleagues (Shek 2006; Shek and Ma 2006) launched a project entitled “P.A.T.H.S. to Adulthood: A Jockey Club Youth Enhancement Scheme” (Project P.A.T.H.S.), which has contributed to our understanding of PYD by taking a different framework other than the Five Cs. This project included a prevention program aimed at fostering positive youth development of local adolescents (Shek and Ma 2006) and a 6-year longitudinal study tracking the development of personal and family well-being of adolescents over the high school years (Shek and Lin 2016). When measuring PYD, this project used the framework proposed by Catalano et al. (2004).

Based on a review of 77 positive youth development programs in the United States, Catalano et al. (2004) concluded that there were 25 successful programs. They also identified 15 constructs intrinsic to successful programs, including bonding, social competence, emotional competence, cognitive competence, behavioral competence, moral competence, resilience, self-determination, spirituality, self-efficacy, clear and positive identity, belief in the future, and prosocial norms, recognition for positive behavior, and opportunity for prosocial involvement. Accordingly, Shek and his colleagues conceptualized and measured positive youth development in terms of these 15 constructs (Shek et al. 2007; Shek and Ma 2010). In the Project P.A.T.H.S., Catalano’s framework can also be understood in terms of “Five Cs”, including competence (i.e., social competence, emotional competence, cognitive competence, and behavioral competence), confidence (i.e., self-efficacy), caring (i.e., prosocial norms, recognition for positive behavior, and opportunity for prosocial involvement), connection (i.e., bonding), and character (i.e., moral competence). Additionally, this framework includes other PYD attributes such as spirituality, resilience, and belief in the future, which are also critical to helping youth thrive (e.g., Callina et al. 2015; Fergus and Zimmerman 2005; Shek and Wu 2013). The 15 PYD constructs are described below:

1. Bonding (BO): having positive relationships with healthy adults and peers.
2. Resilience (RE): being able to cope with and bounce back from adversity.
3. Social competence (SC): having the interpersonal skills that facilitate effective interaction with others.
4. Emotional competence (EC): having the ability to deal with emotions of self and others.
5. Cognitive competence (CC): having the intellectual ability to solve problems.
6. Behavioral competence (BC): having the ability to take action.
7. Moral competence (MC): having the ability to differentiate right and wrong and do good.
8. Self-determination (SD): being able to think independently and having a sense of autonomy.
9. Self-efficacy (SE): having the skills for coping and mastery.
10. Spirituality (SP): having a sense of purpose in life, hope or beliefs in a higher power.
11. Beliefs in the future (BF): having the ability to develop future possible goals and develop an optimistic outlook.
12. Clear and positive identity (SI): developing a healthy identity and self-image.
13. Prosocial involvement (PI): making a contribution to groups and communities.
14. Prosocial norms (PN): developing clear standards for prosocial engagement.
15. Recognition for positive behavior (PB): having contexts that recognize and reinforce positive behavior.

The initial phase of the Project P.A.T.H.S. generated evidence on the developmental trajectories of the PYD attributes. To illustrate, in Shek and Ma’s (2012) study with eight waves of assessments from Grade 7 to Grade 11, the PYD attributes (i.e., moral competence, behavioral competence, clear and positive identity) declined in the early high school years among the students who participated in the Project P.A.T.H.S. and also their non-participating counterparts. Despite the participating students showing a faster decline in the first year of high school than the non-participating students, their PYD attributes grew faster in their later years of high school. The trajectories of adolescent ill-welling indicated by risk behavior were also documented by Shek and Yu (2012). During the same period of time, adolescents’ substance abuse and problem behavior intention increased but with the growth slowing down over time.

In the 6-year longitudinal study in the Project P.A.T.H.S., a few preliminary studies have shown that emotional competence increased with growth rate diminishing over time (Shek and Leung 2016); social competence declined at a decreasing rate over time (Shek et al. 2016b); cognitive competence (Shek and Pu 2016) and moral competence (Shek and Liang 2016a, b) increased steadily while resilience decreased at a constant rate (Shek et al. 2016a); self-efficacy remained invariant (Shek and Liang 2016a, b).

### Limitations of Previous Studies

The findings derived from the 4-H study are insightful for constructing PYD theory as well as improving the design and implementation of PYD program. However, the scientific understanding based on one single sample pool, notwithstanding large, is apparently limited. In particular, cultural variation may exist in the developmental change of PYD, as previous research has suggested ethnic or cultural variations in the developmental trajectories of many of the attributes related to personal well-being. To illustrate, in Moneta et al. (2001)’s study, global self-esteem increased from Grade 6 to Grade 12 in the European Americans, African Americans, and Asian Americans, but showed an inverted U-shape of change in Hispanic Americans; “feeling successful” showed an inverted U-shape of change in Asian Americans but a U-shape of change in other ethnic groups. Additionally, the intrinsic academic motivation of American adolescents remained unchanged at a relatively low level (vs. Chinese adolescents), whereas that of Chinese adolescents decreased between Grade 7 and Grade 8 (Wang and Pomerantz 2009). Taken together, to advance our understanding of positive youth development, a rigorous replication and extension with a non-Western sample is strongly needed.

Furthermore, previous studies are limited in the PYD attributes employed. Most of the previous studies were conducted under the 5Cs framework, while many other positive youth development attributes were left under-examined. As mentioned above, Shek and colleagues adopted Catalano et al.’s framework and extended the examination of the additional PYD attributes. However, more attributes such as spirituality and belief in the future, which are critical to adolescent development are yet to be investigated (Callina et al. 2015; Shek and Wu 2013). It is also important to understand the developmental patterns of more PYD attributes.

### The Current Study

With regard to these research gaps, the major objective of the current study was to understand the developmental changes in personal well-being defined by PYD attributes over the adolescence. This objective was met by replicating and extending previous studies via using the framework of Catalano et al. (2004) and adopting a longitudinal design with six waves of assessments.

Our hypotheses were made based on the PYD approach as well as the unique features of the Chinese adolescents in Hong Kong. Similar to the US adolescents, Chinese adolescents have to go through transformation in individual, relationships and developmental contexts, particularly the mismatch between individual developmental needs and contexts, which increases adolescents’ vulnerability to damped personal wellbeing (Liu and Xin 2015). Moreover, Chinese adolescents in Hong Kong face two additional traditional stressors, academic stress and parental control. It is well-known that there is a morbid emphasis on academic excellence in Chinese societies such as Hong Kong (Lee et al. 2006; Shek 2005), with academic stress found to impair personal well-being (e.g., Lee et al. 2006; Quach et al. 2015). While academic competitiveness is fierce in school, Chinese parents’ expectation on children probably adds to the academic pressure. Chinese parental expectation on children is not oriented to well-being, but instead to academic achievement, good conduct, and filial piety (Shek and Chan 1999). Parenting that aims at boosting academic success through strong psychological control and heightened attention on academic failure may have emotional cost among adolescents (Pomerantz et al. 2014). As academic achievement becomes more important when children enter into high school, such stressors probably become larger. However, as predicted by PYD approach, adolescents might be able to overcome the life difficulties and successfully transit into adulthood. Previous studies in Hong Kong also showed a U-shape of PYD development (Shek and Ma 2012). Therefore, upholding the principle of plasticity, we expected a general decline in PYD in the early years of high school yet with a rebound in the late years (i.e., U-shape trajectory).

Nonetheless, as far as the singular PYD attributes were concerned, developmental pattern may vary according to the attribute. For example, considering that academic burden probably increases and the normative evaluation becomes more significant with the coming of entrance examination to college, it is uncertain that whether the PYD attributes related to the academic pressure would rebound. As academic performance is crucial for the evaluation of a Chinese student and closely related to his/her prospect, adolescents’ self-efficacy and belief in the future are possibly contingent on their academic performance (cf. Liao and Wei 2014). Increased normative evaluation may dampen students’ self-efficacy (Schunk and Meece 2005), and excessive academic demands might evoke students’ hopeless feelings about their future (Salmela-Aro and Upadyaya 2014). Accordingly, we expected self-efficacy and belief in the future to decline over the whole high school period. However, it is noted that given the limited literature about the trajectories of different PYD attributes, our investigation was exploratory in nature.

## Method

### Participants and Procedure

Participants consisted of 3,328 grade 7 students (Mage = 12.59 ± 0.74 years; 47.2 % female) from 28 local high schools in Hong Kong. These participants were assessed annually from Grade 7 to Grade 12 with an exception of Grade 12. As they needed to sit for the public examination, the interval of Grade 11 and Grade 12 was shortened to be about 10 months. The attribution rates of each wave ranged from 12.7 to 28.3 % due to dropout, transferring school, or absence on the assessment day. A total of 2,023 adolescents completed all six waves of assessments, of which 51.4 % (*n* = 1,040) were females, 6.4 % (*n* = 129) received government subsidy due to poverty, and 80.9 % lived in intact families with two biological parents. Adolescents who completed all six waves of assessments performed slightly better than those with incomplete assessments in some PYD attributes, including BO, RE, SC, PB, EC, BC, MC, PI, PN, SP, *t*s >2.45, *d*s = .086–.184. As the individual growth curve modelling excluded the adolescents with incomplete assessment data, the interpretation of our results needs caution.

The participants responded in a classroom setting during school hours to a battery of questionnaires about their personal and family well-being. Parental consent and school consent had been obtained before the administration of the project, while individual informed consent had been obtained at each wave of assessment. A trained research assistant was present in the classroom to administrate the survey and answer the questions.

### Instruments

Positive youth development was evaluated by the short-version of Chinese Positive Youth Development Scale (CPYDS; Shek et al. 2007; Sun and Shek 2013). According to Catalano et al. (2004)’s proposal, the CPYDS assesses 15 PYD constructs, including social competence (SC), moral competence (MC), emotional competence (EC), behavioral competence (BC), cognitive competence (CC), recognition for positive behavior (PB), self-efficacy (SE), self-determination (SD), bonding (BO), resilience (RE), clear and positive identity (SI), beliefs in the future (BF), prosocial involvement (PI), prosocial norms (PN), and spirituality (SP). Each construct was assessed by two to three items on a 6-point scale except spirituality (7-point scale). Shek and Ma (2010) showed that four higher-order components could be extracted from these constructs, namely, cognitive-behavioral competence (i.e., CC, BC, SD), prosocial attributes (i.e., PI, PN), positive identity (i.e., SI, BF), general positive youth development attributes (i.e., BO, RE, SC, PB, EC, MC, SE, SP). The reliabilities of total scale and subscales except SE and PN exceeded the widely accepted .70 coefficient Cronbach’s alpha standard (Coaley 2010; Kline 2013) (see Tables 3 and 4), which indicated good internal consistency. In the PN subscale, two items measured the prosocial norm in terms of doing good, while the third item measured it in terms of obeying school regulations. Adding the third item probably lowered the coefficient alpha. However, all of the three items were conceptually congruent, and thus we did not drop the third item for a higher reliability. The low coefficient alpha of the SE subscale may be due to the inclusion of two items alone. Considering that the reliabilities exceeded .60, we still analyzed them as much previous research did (e.g., Lam et al. 2010; Rudolph et al. 2001). Yet, lower internal consistency may lead to an underestimated effect. Caution is warranted in generalizing the results of these two PYD attributes.

**Table 3:** Descriptive statistics of positive youth development variables across 6 waves (total scale and higher-order factors)

	Mean (SD)						Reliability					
	Grade 7	Grade 8	Grade 9	Grade 10	Grade 11	Grade 12	Grade 7	Grade 8	Grade 9	Grade 10	Grade 11	Grade 12
PYD	4.51 (.70)	4.45 (.68)	4.46 (.64)	4.47 (.60)	4.45 (.58)	4.48 (.58)	.93	.96	.93	.96	.96	.96
Higher-order factors												
CBC	4.45 (.75)	4.43 (.73)	4.44 (.68)	4.46 (.64)	4.43 (.63)	4.47 (.62)	.87	.89	.89	.89	.88	.88
PA	4.50 (.89)	4.42 (.85)	4.42 (.81)	4.45 (.78)	4.42 (.75)	4.47 (.74)	.83	.83	.83	.84	.81	.81
PI	4.24 (.96)	4.19 (.95)	4.17 (.92)	4.14 (.88)	4.09 (.85)	4.14 (.85)	.87	.89	.89	.88	.87	.86
GPYDQ	4.58 (.71)	4.53 (.69)	4.54 (.65)	4.57 (.62)	4.54 (.60)	4.57 (.60)	.93	.93	.93	.93	.93	.93

*PYD* Positive youth development total scale, *CBC* Cognitive-behavioral competence, *PA* Positive attributes, *PIT* Positive identity, *GPYDQ* General positive youth development attributes

**Table 4:** Descriptive statistics of positive youth development variables across 6 waves (individual constructs)

	Mean (SD)						Reliability					
	Grade 7	Grade 8	Grade 9	Grade 10	Grade 11	Grade 12	Grade 7	Grade 8	Grade 9	Grade 10	Grade 11	Grade 12
BO	4.70 (.88)	4.62 (.87)	4.62 (.83)	4.70 (.77)	4.68 (.74)	4.72 (.74)	.74	.76	.78	.77	.77	.78
RE	4.64 (.91)	4.59 (.87)	4.62 (.82)	4.61 (.79)	4.60 (.78)	4.60 (.77)	.79	.81	.81	.81	.82	.83
SC	4.74 (.89)	4.68 (.87)	4.64 (.82)	4.65 (.78)	4.65 (.77)	4.65 (.76)	.86	.88	.88	.88	.88	.88
PB	4.33 (.98)	4.23 (.94)	4.21 (.89)	4.30 (.82)	4.25 (.83)	4.31 (.81)	.76	.77	.78	.79	.81	.81
EC	4.26 (.95)	4.30 (.88)	4.32 (.85)	4.38 (.79)	4.36 (.80)	4.38 (.78)	.73	.74	.75	.74	.77	.78
CC	4.32 (.91)	4.36 (.85)	4.39 (.80)	4.43 (.75)	4.40 (.73)	4.47 (.72)	.81	.83	.84	.82	.82	.82
BC	4.54 (.83)	4.51 (.79)	4.51 (.74)	4.54 (.69)	4.51 (.70)	4.56 (.69)	.71	.76	.76	.74	.73	.74
MC	4.37 (.91)	4.41 (.85)	4.46 (.81)	4.49 (.80)	4.50 (.75)	4.55 (.74)	.73	.75	.74	.75	.70	.70
SD	4.47 (.89)	4.43 (.85)	4.43 (.81)	4.41 (.78)	4.37 (.76)	4.41 (.76)	.75	.77	.77	.77	.77	.79
SE	4.35 (.93)	4.35 (.91)	4.40 (.86)	4.40 (.83)	4.37 (.81)	4.43 (.79)	.65	.66	.64	.64	.61	.61
SI	4.09 (1.03)	4.08 (1.00)	4.10 (.95)	4.11 (.91)	4.10 (.88)	4.16 (.88)	.78	.80	.80	.80	.79	.79
BF	4.39 (1.05)	4.30 (1.04)	4.25 (1.02)	4.16 (.99)	4.08 (.96)	4.12 (.95)	.84	.85	.85	.83	.79	.78
PI	4.37 (1.04)	4.29 (.98)	4.33 (.93)	4.33 (.89)	4.31 (.86)	4.40 (.88)	.80	.82	.83	.82	.81	.83
PN	4.64 (.96)	4.54 (.93)	4.51 (.88)	4.56 (.86)	4.52 (.84)	4.53 (.81)	.72	.72	.69	.72	.69	.68
SP	5.14 (1.32)	5.01 (1.29)	5.01 (1.26)	4.96 (1.23)	4.92 (1.24)	4.95 (1.23)	.88	.89	.90	.92	.91	.92

*BO* Bonding, *RE* Resilience, *SC* Social Competence, *PB* Recognition for Positive Behavior, *EC* Emotional Competence, *CC* Cognitive Competence, *BC* Behavioral Competence, *MC* Moral Competence, *SD* Self-Determination, *SE* Self-Efficacy, *SI* Clear and Positive Identity, *BF* Beliefs in the Future, *PI* Prosocial Involvement, *PN* Prosocial Norms, *SP* Spirituality

### Data Analysis Plan

Individual growth curve modeling (IGC) was adopted to estimate the developmental trajectories of positive youth development constructs. IGC allows researchers to capture an individual’s developmental change in a variable over time across multiple time points as well as the inter-individual differences in the development change, and it can easily model multi-wave assessments with different lengths of the intervals (Hox and Boom 2012). It has been widely used in previous studies documenting the developmental trajectories of attributes over time (e.g., Gutman et al. 2003; Shek and Yu 2012). For the current study, as we were interested in looking at the general developmental patterns, we only examined the intra-individual developmental change of positive youth development attributes over the period of assessment without examining inter-individual predictors.

Three steps were employed to understand the developmental change. First, an intercept-only model (Model 1) was estimated, which served as a baseline model. Second, an unconditional growth model with linear slope (Model 2) was conducted. This model estimated the extent to which the construct increased or decreased over time. Third, an unconditional growth model with both linear and quadratic slopes was conducted (Model 3). This model estimated the extent to which the change was curved or bent. To show the incremental value of quadratic slope, proportional reduction in variance (PRV, Model 2 vs. Model 3; Singer and Willett 2003) was reported for the significant quadratic changes. Model fit was evaluated by three indices that have been commonly used in previous studies (Shek and Yu 2012; Wray‐Lake et al. 2010), including 2log likelihood (i.e., likelihood ratio test), Akaike Information Criterion (AIC), and Bayesian Information Criterion (BIC). The smaller the number, the better the model fit. IGC was modeled using Linear Mixed Model (LMM) in SPSS 22.0 statistical software (IBM SPSS Statistics, IBM Corp, Somers, NY). The effect sizes of the significant changes were reported in terms of Cohen’s d (Cohen 1992).

## Results

**Fig. 1:**
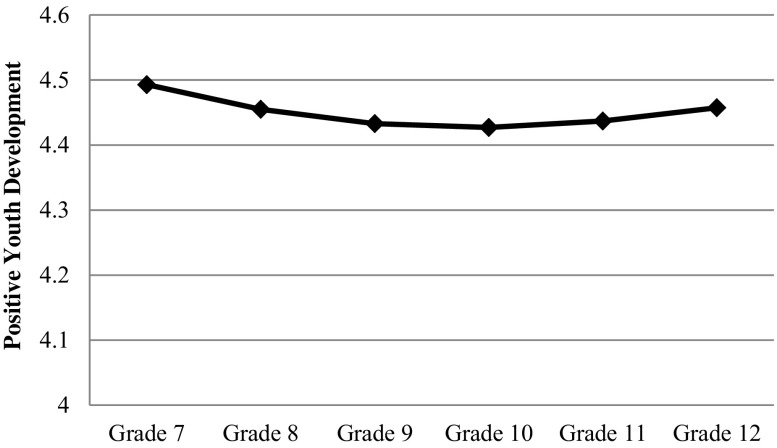
Average developmental change of positive youth development

**Fig. 2:**
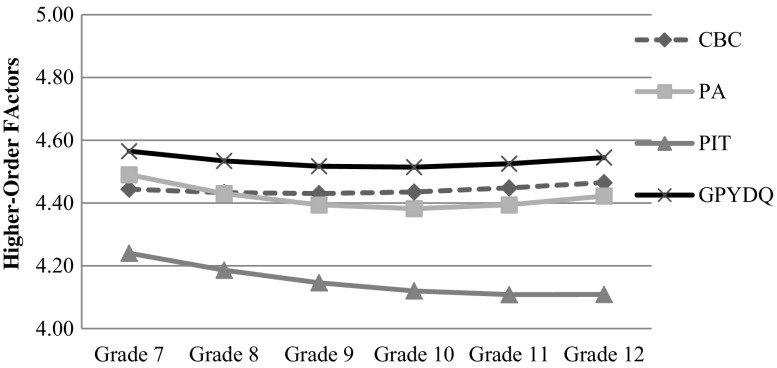
Average developmental changes of higher-order factors. *CBC* Cognitive-behavioral competence, *PA* Positive attributes, *PIT* Positive identity, *GPYDQ* General positive youth development

**Fig. 3:**
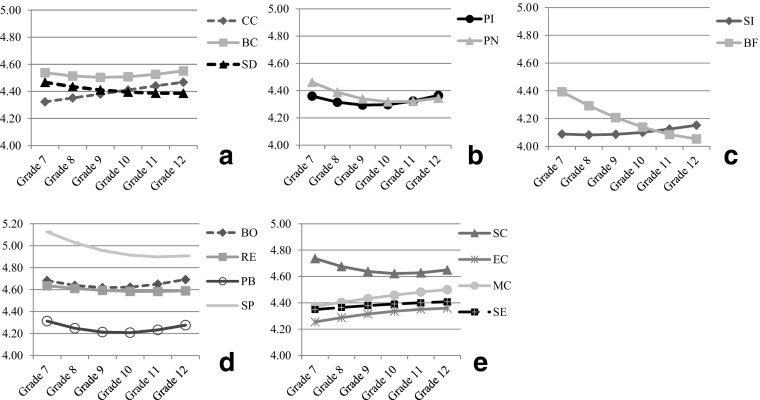
Average developmental changes of the individual constructs. *Note*s: cognitive-behavioral competence (**a**), prosocial attributes (**b**), positive identity (**c**), four subscales of general positive youth development attributes (**d**), and another four subscales of general positive youth development attributes (**e**); *CC* Cognitive Competence, *BC* Behavioral Competence, *SD* Self-Determination, *SI* Clear and Positive Identity, *BF* Beliefs in the Future, *PI* Prosocial Involvement, *PN* Prosocial Norms, *BO* Bonding, *RE* Resilience, *SC* Social Competence, *PB* Recognition for Positive Behavior, *EC* Emotional Competence, *MC* Moral Competence, *SE* Self-Efficacy, *SP* Spirituality

The level and shape of the developmental trajectory of the positive youth development total score, four higher-order factors (i.e., cognitive-behavioral competence; prosocial attributes; positive identity; general positive youth development attributes), and 15 constructs were modeled by IGC. The developmental changes were plotted and shown in Figs. 1, 2 and 3. Model 3 fitted the data better than Model 1 (Δ*χ* ^2^_(7)_s > 333.62) and Model 2 (Δ*χ* ^2^_(4)_s > 74.94) across all constructs, *p*s < .001. Thus, the interpretation of results was based on Model 3 (see Tables 5, 6 and 7).

**Table 5:** Unconditional growth models of PYD total score and higher order factors

		PYD		CBC		PA		PIT		GPYDQ	
		Estimate	SE	Estimate	SE	Estimate	SE	Estimate	SE	Estimate	SE
Fixed effects											
Intercept	*β*_*0j*_										
Intercept	*γ*_*00*_	4.493c	.013	4.444c	.013	4.490c	.015	4.240c	.017	4.565c	.012
Linear slope	*β*_*1j*_										
Time	*γ*_*10*_	−.046c	.008	−.015	.009	−.072c	.010	−.061c	.011	−.038c	.008
Quadratic slope	*β*_*2j*_										
Time^2^	*γ*_*20*_	.008c	.001	.004a	.002	.012c	.002	.007b	.002	.007c	.002
Random effects											
Level 1 (within)											
Residual	*r*_*ij*_	.112c	.002	.189c	.003	.254c	.004	.275b	.005	.123c	.002
Level 2 (between)											
Intercept	*u*_*0j*_	.381c	.013	.373c	.014	.521c	.019	.659c	.023	.384c	.013
Time	*u*_*1j*_	.064c	.005	.064c	.007	.108c	.010	.108c	.010	.072c	.005
Time^2^	*u*_*2j*_	.002c	.000	.002c	.000	.003c	.000	.003c	.000	.002c	.000
Fit statistics											
Deviance		20627.799		27442.065		33474.337		35460.865		22720.234	
AIC		20647.799		27462.065		33494.337		35480.865		22740.234	
BIC		20723.857		27538.859		33105.147		35557.897		22816.827	
df		10		10		10		10		10	
PVR^r^		11.3 %		6.4 %		9.4 %		7.8 %		11.5 %	

*PYD* Positive youth development total scale, *CBC* Cognitive−behavioral competence, *PA* Positive attributes, *PIT* Positive identity, *GPYDQ* General positive youth development, ^*r*^ proportional reduction in variance, the proportion of incremental variance explained by quadratic model relative to linear model; c: *p* < .001; b: *p* < .01; a: *p* < .05

**Table 6:** Unconditional growth models of constructs of CBC, PA and PIT

		CC		BC		SD		PI		PN		SI		BF	
		Estimate	SE	Estimate	SE	Estimate	SE	Estimate	SE	Estimate	SE	Estimate	SE	Estimate	SE
Fixed effects															
Intercept	*β*_*0j*_														
Intercept	*γ*_*00*_	4.323c	.015	4.538c	.014	4.467c	.015	4.360c	017	4.462c	.016	4.088c	018	4.393c	.018
Linear slope	*β*_*1j*_														
Time	*γ*_*10*_	.028a	.011	−.031b	.010	−.036b	.011	−.057b	.013	−.087c	.011	−.011	.012	−.109c	.012
Quadratic slope	*β*_*2j*_														
Time^2^	*γ*_*20*_	−.0004	.002	.007b	.002	.004	.002	.012b	.003	.013c	.002	.005a	.002	.008b	.002
Random effects															
Level 1 (within)															
Residual	*r*_*ij*_	.313	.005	.294c	.005	.303c	.005	.405c	.007	.307c	.005	.332c	.005	.385c	.006
Level 2 (between)															
Intercept	*u*_*0j*_	.496c	.019	.389c	.017	.465c	.185	.634c	.025	.598c	.022	.727c	.026	.740c	.027
Time	*u*_*1j*_	.097c	.011	.086c	.010	.085c	010	.159c	.015	.117c	.011	.126c	.011	.129c	.013
Time^2^	*u*_*2j*_	.002c	.000	.002c	.000	.002c	.004	.005c	.001	.003c	.000	.003c	.000	.003c	.000
Fit statistics															
Deviance		35217.424		33582.599		34741.952		40238.569		36650.391		38225.545		40343.985	
AIC		35237.424		33602.599		34761.952		40258.569		36670.391		38245.545		40363.985	
BIC		35314.528		33679.675		34838.920		40335.685		36747.514		38322.652		40441.093	
df		10		10		10		10		10		10		10	
PVR^r^		−		6.0 %		−		13.8 %		31.1 %		7.4 %		6.9 %	

*CBC* Cognitive-behavioral competence, *PA* Positive attributes, *PIT* Positive identity, *CC* Cognitive Competence, *BC* Behavioral Competence, *SD* Self-Determination, *SI* Clear and Positive Identity, *BF* Beliefs in the Future, *PI* Prosocial Involvement, *PN* Prosocial Norms, ^*r*^ proportional reduction in variance, the proportion of incremental variance explained by quadratic model relative to linear model; c: *p* < .001; b: *p* < .01; a: *p* < .05

**Table 7:** Unconditional growth models of constructs of GPYDQ

		BO		RE		SC		PB		EC		SE		SP		MC	
		Estimate	SE	Estimate	SE	Estimate	SE	Estimate	SE	Estimate	SE	Estimate	SE	Estimate	SE	Estimate	SE
Fixed effects																	
Intercept	*β*_*0j*_																
Intercept	*γ*_*00*_	4.682c	.015	4.635c	.015	4.736c	.015	4.313c	.017	4.255c	.016	4.349c	.015	5.126c	.023	4.374c	.015
Linear slope	*β*_*1j*_																
Time	*γ*_*10*_	−.056c	.010	−.029b	.011	−.071c	.011	−.080c	.012	.036b	.011	.017	.012	−.113c	.016	.031b	011
Quadratic slope	*β*_*2j*_																
Time^2^	*γ*_*20*_	.012c	.002	.004	.002	.011c	.002	.015c	.002	−.003	.002	−.001	.002	.014c	.003	−.001	.002
Random effects																	
Level 1 (within)																	
Residual	*r*_*ij*_	.273	.004	.304	.005	.283	005	.340	.006	.339	.006	.416ba	.007	.546c	.009	.295	.005
Level 2 (between)																	
Intercept	*u*_*0j*_	.499c	.019	.518c	.020	.510c	.019	.614c	.023	.540c	021	.443c	.020	1.205c	.042	.502c	.019
Time	*u*_*1j*_	.083c	.009	.104c	011	.124c	.011	.124c	.012	.101c	.011	.088c	.013	.268c	.021	.106c	.010
Time^2^	*u*_*2j*_	.002c	.000	.002c	.000	.003c	.000	.003c	.000	.002c	.000	.002c	.000	.007c	.001	.003c	.004
Fit statistics																	
Deviance		34291.698		35574.301		35043.647		37680.918		36794.740		38640.193		47164.437		34668.580	
AIC		34311.698		35594.301		35063.647		37700.918		36814.740		38660.193		47184.437		34688.580	
BIC		34388.834		35671.415		35140.772		37778.041		36891.856		38737.329		47261.525		34765.612	
df		10		10		10		10		10		10		10		10	
PVR^r^		6.4 %		–		9.4 %		7.7 %		–		–		49.4 %		–	

*GPYDQ* General positive youth development, *BO* Bonding, *RE* Resilience, *SC* Social Competence, *PB* Recognition for Positive Behavior, *EC* Emotional Competence, *MC* Moral Competence, *SE* Self-Efficacy, *SP* Spirituality, ^*r*^ proportional reduction in variance, the proportion of incremental variance explained by quadratic model relative to linear model; c: *p* < .001; b: *p* < .01; a: *p* < .05

Generally speaking, the average developmental trajectory of positive youth development included a minor decrease (*d* = −.225) while the decrease slowed down over the 6 years (*d* = .208) (see Fig. 1). The quadratic model was more descriptive of the trajectory than the linear model, explaining 11.3 % more of the variance. It firstly declined but increased again after Grade 10. In fact, a similar developmental trajectory was observed in different constructs based on the higher-order factors, although variation in the discrete subscales was observed.

Regarding cognitive-behavioral competence, the average developmental trajectory was slightly concave (*d* = .082) yet the downward trend was not statistically significant. The quadratic model explained 6.4 % more of the variance relative to the linear model. As shown in Fig. 2, cognitive-behavioral competence reached the lowest point at Grade 9. When looking into the three subscales, we found that behavioral competence (*d* = −.108) and self-determination (*d* = −.125) decreased over time with the decrease of behavioral competence (*d* = .123) but not self-determination slowing down over time (Fig. 3). The turning point of the change of behavioral competence rested at Grade 9, after which behavioral competence increased. In contrast, cognitive competence increased over time without any quadratic change.

Regarding prosocial attributes, the average developmental trajectory included a minor decrease (*d* = −.261) while the decrease slowed down (*d* = .235) over the 6 years (see Fig. 2). The quadratic model explained 9.4 % more of the variance. The trajectory initially decreased yet increased again after Grade 10. Both prosocial involvement (*d*s = −.165 and .177) and prosocial norms (*d*s = −.291 and .225) showed this developmental pattern (see Fig. 3).

Regarding positive identity, the average developmental trajectory included a minor decrease (*d* = −.211) while the decrease slowed down (*d* = .121) over the 6 years. The quadratic model explained 7.8 % more of the variance. As shown in Fig. 2, the declining trend of positive identity was reversed after Grade 11. This developmental pattern was dominated by belief in the future, which kept declining over the whole high school period (*d* = −.324). Clear and positive identity showed a significant quadratic change (*d* = .077), which was due to a small decline from Grade 7 to Grade 8, followed by an increasing trend. Yet, the linear change of clear and positive identity over the 6 years was not statistically significant.

Regarding the general positive youth development attributes, the average developmental trajectory included a minor decrease (*d* = −.180) while the decrease slowed down (*d* = .179) over the 6 years. The quadratic model explained 11.5 % more of the variance. As shown in Fig. 2, the trajectory initially decreased but increased again after Grade 10. This developmental pattern was observed in bonding (*d*s = −.204 and .234), social competence (*d*s = −.240 and .189), recognition for positive behavior (*d*s = −.250 and .259), and spirituality (*d*s = −.266 and .174), and their turning points were at Grade 11, Grade 10, Grade 10, and Grade 11, respectively. In contrast, a constant decline was observed in resilience (*d* = −.097) while no significant change was found in self-efficacy; emotional competence (*d* = .117) and moral competence (*d* = .106) showed a constant increase without any quadratic changes over time.

In short, when the omnibus measure and four composite measures based on the higher-order factors were used, the developmental trajectories showed a U-shape pattern. Besides, four patterns were observed for the discrete subscales: a) adolescents experienced a U-shape development in eight positive youth development constructs, including BO, SC, PB, BC, SI, PI, PN, SP; b) a downward trend over time in three constructs, including a constant decline in RE and SD and a decline with deceleration in BF; c) a constant increase in CC, EC and MC; and d) a stable trend in SE. All the changes were regarded as small (Cohen 1992). When controlling gender, economic status (i.e., receiving government subsidy or not), and family intactness in the models, the results were largely the same except three cases. First, the quadratic change of CBC changed from significant to marginally significant (*β*_20_ = .003, *SE* = .002, *p* = .075). Second, the quadratic change of SD changed from insignificant to significant (*β*_20_ = .005, *SE* = .002, *p* = .036). Third, the quadratic change of SI changed from significant to marginally significant (*β*_20_ = .005, *SE* = .002, *p* = .055). All the variations of the mean intercept, mean linear slopes, and mean quadratic slopes were significant. This indicated that there were individual differences in the developmental trajectories of PYD attributes (see Tables 5, 6 and 7).

## Discussion

The overarching goal of the current study was to examine the developmental trajectories of positive youth development attributes over high school years to see whether there was any support for the PYD approach. As expected, a U-shape curve was observed in the composite PYD attributes (i.e., PYD total score, CBC, PA, PIT, and GPYDQ) with a drop in the early adolescence followed by a rebound or a slower drop in the late adolescence. Although these changes were small, the findings are inconsistent with the “storm and stress” view that expects constant “turbulence” in adolescence (Hall 1904). However, when analyzed separately, variations were found across different PYD attributes, with some following a typical U-shape curve while others showing an increasing, a declining, or a constant trend. Considering that the schools of our sample were randomly selected from the Hong Kong high schools, our sample was to a certain degree representative of adolescents who are receiving formal schooling.

Compared with the results from the 4-H study, we found similarities and differences in the developmental trajectories of PYD attributes. First, similar to the findings of the 4-H study that showed competence and connection declining in early high school years (Geldhof et al. 2014), we found that social competence, behavioral competence and bonding dropped among Hong Kong adolescents in the early high school years yet with a rebound later. In contrast, we found that cognitive competence, emotional competence and moral competence increased during the high school years. These findings suggest that different competences might show divergent developmental trajectories, and thus future studies should consider them separately. In addition, it implies further effort is needed to identify why an upward trend was found for some competences while a downward trend occurs in others.

Dissimilar to Hilliard et al. (2014)’s findings of an increase in adolescent prosocial behavior towards others and the community, our findings suggest that prosocial attributes of Hong Kong adolescents declined in the first few years of high school followed by an increase in the later years. This result is understandable because in the senior high school years, students have to fulfill the requirement of “Other Learning Experiences” (OLE; Education Commission 2000), which includes community service. This provides students with extrinsic motivation to participate in voluntary work. However, this speculation needs further confirmation. It is noteworthy that there is mounting evidence pointing out the benefits of prosocial behavior for adolescent development (for a review, see Kuperminc et al. 2001). For example, adolescents who engaged in community service had better academic adjustment, less problem behavior, and more civic efficacy and knowledge, no matter whether it is due to school requirement or personal wishes (Schmidt et al. 2007). Therefore, we recommend different parties (e.g., school, parents, and government agencies) to encourage prosocial involvement among young people and nurture their prosocial behavior.

Moreover, Hong Kong adolescents’ moral competence increased steadily over the high school years, which is different from the stable trend of character among the US adolescents in the 4-H study (Geldhof et al. 2014). Character pertains to a set of good traits that drive people to pursue the good while moral competence refers to the ability to do the good (Park and Peterson 2006). It is possible that with age, adolescents develop greater ability to translate their moral intention into behavior. Due to different measures and methodologies used in the 4-H study and Project P.A.T.H.S., the comparisons above warrant caution. For a better understanding of the variations in PYD trajectories across adolescents from different cultural contexts, studies with comparable sample, same methods and same methodologies should be used in the future (see Wang and Pomerantz 2009).

We originally expected that self-efficacy and belief in the future would keep declining as they may be sensitive to academic pressure, yet this was not true for self-efficacy. Hong Kong adolescents showed stable beliefs about their overall capacity notwithstanding increasing academic pressure. Previous findings of the developmental pattern of self-efficacy over adolescents are not congruent with some documenting a downward trend while others reporting an upward trend (for a review, see Schunk and Meece 2005). Therefore, we need to further examine the protective factors that are unique to Hong Kong adolescents, such as heightened teacher support and student support (Jia et al. 2009). However, the Hong Kong adolescents’ belief in future did decline over the whole high school year. When adolescents harbor hope or have a more optimistic outlook about their future, they are more likely to have better adjustment such as better personal well-being and less engagement in risk behavior (Carvajal et al. 1998; Wong and Lim 2009). Therefore, this enduring deterioration deserves more attention of parents and school.

Overall, the current results unfolded a positive developmental pattern during adolescence, which lends support to PYD approach. Meanwhile, these findings also provide implications for the further refining of the PYD theory. First, consistent with previous findings of the 4-H study (e.g., Geldhof et al. 2014), most of the Hong Kong adolescents maintained medium-to-high levels of PYD (all of the mean scores over 4) across grades.

Second, consistent with the previous literature on adolescent well-being, including self-esteem (Rhodes et al. 2004) and life satisfaction (Shek and Liu 2014), our study found that multiple PYD attributes declined over high school years, particularly in the early years. Such changes may reflect a poor adjustment between individual and the context. For example, the parent–child conflicts increase due to the discrepancy between their increasing developmental need for autonomy and their family context where parents still exert a high degree of control, particularly, over academic performance. Also, early adolescents may not adjust to the high school environment, where, compared to primary school, evaluation becomes stricter, performance rather than mastery is emphasized, and competition among peers becomes stronger (Molloy et al. 2011). This is typically the case in Hong Kong (e.g., Lau 2009).

Besides the regulation of the individual-context relations, we may need to take into account the intrapersonal factor, which is the maturity of cognitive functioning, such as abstract thinking and social comparison. Adolescents may adjust their self-understanding from an optimistic one to a realistic one (Eccles et al. 1993) and thus they may experience a negative change of self-appraisal in early adolescence. We did find that cognitive competence increased constantly over time. These findings imply that future studies need to consider not only the interplay between individual and context (e.g., school and family) in the developmental trajectories of PYD attributes, but also the relationships among the different PYD attributes. For example, it is theoretically and practically important to understand how cognitive competence is related to the drop in the belief in the future.

On the other hand, it is important to note that such declining trends were not drastic enough to largely disturb adolescents’ development of strengths, as the effect sizes of the changes were small. Therefore, future studies are needed to investigate what the protective factors are. Previous studies have suggested that compared to the US adolescents, Chinese adolescents had more positive perception of parent–child relationship (Greenberger et al. 2000) and school (Bear et al. 2016; Jia et al. 2009), which indicates that these contexts are perceived to be more supportive to their positive development. However, given that the effect sizes of developmental changes in the 4-H study were not available, we cannot draw a conclusion that the Chinese adolescents experienced smaller changes than the US adolescents. The validation of such a speculation requires further inquiry.

More importantly, the difficulties of transition can be overcome, as we also found that the declining trend were discontinuous for most of the attributes. The rebound or slower rate of decline in many PYD attributes may be due to gradual adjustment to high school or improved quality of friendship (Way and Greene 2006). These results suggest that adolescents are able to acquire some life skills and develop their strengths, and finally navigate themselves through high school years successfully. This reflects the relative plasticity of adolescents (Lerner 2004). Future studies are needed to identify what kinds of contexts (e.g., school climate) can halt the decline of PYD in the early high school years or facilitate the recovery in the late high school years.

Finally, diversity was found in the developmental changes of different PYD attributes. Besides the declining trend, we found that some PYD attributes maintained at a stable level (SE) and some kept increasing (CC, EC, MC). PYD theories highlighted the over-time changes within intrapersonal development, and the individual differences in intrapersonal developmental trajectories (e.g., Lewin-Bizan et al. 2010; Phelps et al. 2007), but they seldom discussed the variation across different PYD attributes. Our study did not only evidence the change across time of many PYD attributes but also unfold the inconsistency in the developmental trajectories among these PYD attributes. These findings suggest that PYD theory should consider the diversity in terms of variation across different PYD attributes. As suggested by the view of interaction between individual and context, future studies can examine what contextual factors account for different trajectories across attributes. For example, is the curriculum in Hong Kong high schools favorable for the enhancement of moral competence, cognitive competence, and emotional competence that are largely for intrapersonal regulation while not very favorable for the enhancement of social competence that is for interpersonal regulation?

### Practical Implications

The developmental profiles of the PYD attributes found in our study provided practical implications for the educational profession and education policy. It is usually easier for school administrators to boost their students’ academic performance given the standardized curriculum and well-established instruction. In contrast, it is relatively difficult and even confusing for the school administrators to promote their students’ personal well-being partially due to a lack of scientific knowledge of adolescent profiles in a non-academic track. Our current results provide school administrators with a reference that helps them to design specific programs and implement specific measures to enhance their students’ PYD. For example, the drop in multiple of the PYD attributes in early adolescence indicates that additional endeavor should be made to promote PYD attributes in or before the early high school years. In addition, considering the variations in the trajectories across different PYD attributes, the effort in promoting PYD attributes may vary according to the magnitude and duration of dip. For the PYD attributes (e.g., BF, RE, and SD) that keep declining over the whole high school period, continual effort is highly needed to enhance them.

High schools can provide prevention programs that nurture adolescents’ PYD. The prevention programs not only offer them with knowledge and skills needed to meet with increasing challenges over the adolescent period (Roth and Brooks-Gunn 2015), but also serve as a positive context where adolescents can practice life skills, build up positive relationships with healthy peers and adults, obtain social support, refine character and make a contribution to the wellness of others and community (Ramey and Rose‐Krasnor 2012). In addition, they might improve the school context such as teacher-student relationship so as to offer students with a challenging, resourceful, and supportive context which is favorable to the growth of PYD. While successful experience can be borrowed from prior cases that promote PYD (Catalano et al. 2012; Durlak et al. 2011; Shek and Ma 2012), future development of prevention programs in Hong Kong still needs to take into account the normative developmental trajectories of local adolescents.

Furthermore, the current findings deliver a message about the plasticity of adolescents to the educational professionals, youth workers, and policy-makers. As argued by the PYD advocators (Lerner 2004), the intrapersonal changes of PYD attributes over time signify the possibility to promote their PYD. Educational professionals and youth workers should have more trust in the plasticity of adolescents and spend more effort to help adolescents improve their PYD qualities. Policy-makers should develop more measures favorable for enhancing adolescents’ personal strengths, such as allocating more resource to support extracurricular activities that are favorable for PYD, or encouraging schools to incorporate PYD education into formal curriculum.

### Limitations and Future Directions

The current study should be interpreted with several caveats. First, the current study simply focused on the average developmental trajectories of the PYD attributes while leaving the individual differences untested. Although a U-shape pattern was still observed when some demographic variables were controlled in this study, previous studies of project P.A.T.H.S. has suggested that family intactness, economic status, and family dynamics at Grade 7 had effects on the subsequent developmental changes of the PYD attributes (e.g., Shek et al. 2016a; Shek and Liang 2016a, b). The variances of the slopes in our study were statistically significant, which indicates there are factors at an individual level that can explain the variation in the developmental trajectories. In addition, according to previous findings in the 4-H study, not every adolescent follows the same developmental trajectory of PYD (see Table 2). Thus, we call for further studies that account for inter-individual difference. Using a person-centered approach to examine diverse developmental trajectories among Hong Kong adolescents is a missing yet intriguing inquiry.

Second, all study variables were based on adolescents’ self-report. Thus, developmental trajectories reflect the changes of adolescents’ self-appraisal on their own competence, connection, prosocial attributes and other PYD attributes. It may not denote the actual changes of PYD attributes. For a more comprehensive understanding of adolescent developmental pattern in PYD attributes, future studies need to consider other informants such as peers, parents, and teachers and use other methods such as observation. In short, the use of multiple informants can give a more holistic picture about changes in personal well-being in adolescents.

Last but not least, the current sample only included adolescents with formal schooling, while the dropouts were excluded. In Hong Kong, 15-year-old youth are legally allowed to work. Those who discontinue the schooling probably experience a different developmental trajectory (e.g., Janosz et al. 2008). The homogeneity of our sample seemed to increase across time with the underachieving students dropping out. In addition, the individual growth curve modeling excluded the case with incomplete data, which increased the homogeneity of the sample. Our current findings primarily demonstrated the developmental pattern of adolescents who attended the same school for their entire high school time. Thus, the generalizability of the current results is limited. Future studies can consider what kind of developmental trajectory of personal wellbeing will predict dropout from high school.

In summary, despite these limitations, the current study provided the profile of adolescents’ development in personal wellbeing in terms of a large spectrum of PYD attributes over high school years. These findings contributed to the further refinement of the PYD theory and improvement of youth education and policy.
